# Epidemiological and clinical characteristics of pertussis in children and their close contacts in households: A cross-sectional survey in Zhejiang Province, China

**DOI:** 10.3389/fped.2022.976796

**Published:** 2022-08-18

**Authors:** Luo-Na Lin, Jin-Si Zhou, Chun-Zhen Hua, Guan-Nan Bai, Yu-Mei Mi, Ming-Ming Zhou

**Affiliations:** ^1^Department of Infectious Diseases, National Clinical Research Center for Child Health, The Children’s Hospital, Zhejiang University School of Medicine, Hangzhou, China; ^2^Department of Clinical Laboratory, National Clinical Research Center for Child Health, The Children’s Hospital, Zhejiang University School of Medicine, Hangzhou, China

**Keywords:** *Bordetella pertussis*, children, close contacts, bacteria culture, antibiotic resistance

## Abstract

**Background:**

Despite the expanded immunization programs, the “re-emergence of pertussis” has become a global concern in recent years. At present, the prevalence of pertussis in China is seriously underestimated, and the role of close contact on the disease spreading in children remains unclear.

**Objectives:**

Our study aimed to investigate pertussis’s epidemiological and clinical characteristics in children and their close contacts in households, as well as the antimicrobial resistance of *Bordetella pertussis* (*B. pertussis*) in Zhejiang Province, China.

**Methods:**

We have collected the retrospective and prospective data of children who were suspected of pertussis and their close contacts in households from January 1, 2018, to December 31, 2020, in the Children’s Hospital, Zhejiang University School of Medicine, Hangzhou, Zhejiang, China. Nasopharyngeal swabs were collected and cultured for *B. pertussis*. Antibiotics susceptibility test was determined by using E-test methods. Clinical information was collected from the medical records systems.

**Results:**

A total of 350 index patients and their 946 family members (close contacts in households) from 350 families were recruited. *B. pertussis* strains were isolated from 130 (37.1%) index patients and 116 (12.3%) close contacts. 37 index patients had negative culture results for *B. pertussis* while their close contacts were positive. A higher positive rate was found in female adults than that in male adults (16.3% vs. 5.1%, *P* < 0.01). The positive rate in index patients from multi-child families was significantly higher than that from one-child families (51.7% *vs.* 37.7%, *P* < 0.05). 53.3% of the pertussis patients were under 6 months of age. 98 (75.4%) isolates had MICs ≥ 256 mg/L to erythromycin, azithromycin, and clindamycin, and 127 (97.7%) had MICs < 0.016 mg/L to piperacillin.

**Conclusion:**

Infants under 6 months of age are at high risk of pertussis, and close contacts in households are prone to cluster infection. Culture for *B. pertussis* both in children and their close contacts contributes to improving the diagnosis rate of pertussis in children. Isolates of *B. pertussis* in China are highly resistant to macrolides.

## Introduction

Pertussis is a highly contagious, vaccine-preventable acute respiratory infection caused by *Bordetella pertussi*s (*B. pertussis*) and affects all age groups. Since 1947, the global Expanded Program on Immunization (EPI) was executed, and its incidence has been effectively curtailed (1). However, re-emergence has become a global concern in the last decade. Even in countries with a high coverage rate of vaccination, such as the United States, Canada, and the United Kingdom, the number of reported pertussis cases has been increasing (2–4). According to the data from the Chinese Center for Disease Control and Prevention (China CDC), the number of the reported pertussis cases had decreased to less than 3,000 annually during 2006–2013, however, it increased substantially since 2014 and even up to 30,027 in 2019 (5). The real epidemic situation of pertussis in China has been seriously underestimated that warrants further study (6).

The reasons for the resurgence are multifactorial including the waning of vaccine acquired immunity, the difference in vaccination strategies, the adaptation of *B.* pertussis strains, and an increase in disease awareness due to the strengthening of diagnostic sensitivity and surveillance sensitivity (7). Notably, Hewlett and Edwards proposed that the shift in pertussis transmission patterns plays an important role in pertussis resurgence (8). In the prevaccination era, the majority of pertussis cases occurred in children, *B. pertussis* strains are transmitted mainly from children to adolescents and adults. After the pertussis vaccine has been established in a population, *B. pertussis* strains are mainly transmitted from adolescents and adults to infants. Previous studies of seroepidemiology in Chinese population showed that pertussis might be underestimated in adults and adolescents (9). The positivity rate of the IgG antibody against pertussis toxin (PT-IgG) in adults and adolescents in China ranged from 1.6 to 8.60%, and accordingly, the estimated recent/acute pertussis infection rates ranged from 3,926/100, 000 to 13,898/100, 000, which was much higher than the actual annual mean occurrence rate in China (0.34/100,000) (2, 9, 10). The above-mentioned findings were summarized in Supplementary Table 1. Such change in the epidemiology of disease comes to a significant increase in pertussis among adolescents, adults, and older adults, who are now convinced to transmit pertussis to their unvaccinated or partly immunized infants and children (10). Existing pertussis epidemiological data for adolescents, adults, and older adults are limited due to their high incidence of asymptomatic and mild/atypical infection accompanied by the low visiting rate of hospitals, high misdiagnosis rate, and missed diagnosis rate (11). Therefore, studies of pertussis infection in the family unit have attracted global concerns.

A comprehensive understanding of epidemiological characteristics of pertussis infection among close contacts in households can help to diagnose pediatric pertussis, however, the involvement of close contacts in households in the pathogenesis of pertussis is not clear. A prospective study suggested that the increased risk of household transmission was associated with a sibling or child of a primary case, not vaccination or undervaccination and lack of chemoprophylaxis therapy (12). A British study also found that teenagers and adults who had contact with pertussis patients aged 10–14 years old were more likely infected than those who had contact with very young children, for instance, younger than 1 year old (13). Mothers were found to be the main source of pertussis infection compared to other family members in a household with pediatric pertussis cases (14, 15).

A paroxysmal cough and whooping were the typical clinical symptoms of pertussis. Compared with adult patients, pediatric patients tend to develop into more severe cases and with poor prognoses. Previous studies revealed that younger children had worse clinical manifestations (10). In addition, the emergence of antibiotic resistance has evolved into a looming threat to the current worldwide epidemiology of pertussis, especially macrolide resistance in *B. pertussis* (MRBP) has prevailed in mainland China for years (16). Genotypes differ between the macrolide-sensitive and macrolide-resistant strains, and the acquisition of macrolide resistance may be related to changes in specific molecular types (17). Given the fragility of *B. pertussis*, the sensitivity of culture depends on the quality of the specimen, the vaccination status, the duration of the disease and the previous antibiotics therapy. And the culture of *B. pertussis* takes longer time and needs more experience than the polymerase chain reaction (PCR). Thus, PCR is the most commonly used test for pertussis diagnose in the United States and other countries. Meanwhile, culture is not widely applied in pediatric patients, even rarer in household close contacts. Consequently, research on antimicrobial susceptibility and alternative agents based on culture remain scare.

Therefore, we conducted a hospital-based study by collecting the epidemiological, clinical, and laboratory data of pertussis children and their close contacts in households. In particular, we cultured *B. pertussis* for both patients and their close contacts. The present study aimed to investigate the epidemiological and clinical characteristics of pertussis in children and their close contacts in households, as well as the antimicrobial resistance of *B. pertussis* in China.

## Materials and methods

### Study population

We have collected the retrospective and prospective data of children who were suspected of pertussis and their close contacts in households from January 1, 2018, to December 31, 2020, in the Children’s Hospital, Zhejiang University School of Medicine, Hangzhou, China. Pediatric patients with suspected pertussis and their household close contacts were recruited. The inclusion criteria of patients with suspected pertussis should meet the following conditions simultaneously: (1) presenting with persistent cough of at least 2-week duration or suffering from at least one of the symptoms: paroxysmal cough, nocturnal cough, post-tussive vomiting, or whoop (18); (2) living together with family members who were close contacts; (3) all of their close contacts in the household, as well as the pediatric patients, accepting a collection of nasopharyngeal swab samples for *B. pertussis* culture. A household with more than one pediatric patient with pertussis was recorded as one family, and children except the index case were grouped as sister or brother. Nasopharyngeal swabs were collected upon their first admission or at their first visit to the outpatient department. We extracted the information from the electronic medical records regarding demographic characteristics (i.e., age, sex, dates of onset, incubation time of *B. pertussis*, vaccination status), clinical characteristics (i.e., clinical symptoms and signs, laboratory findings, chest radiograph, and antibiotic treatment. Single antibiotic was prescribed for patients, including erythromycin (10 mg/kg q8h, Hunan Kelun, China), azithromycin (10 mg/kg q24h, Pfizer, Dalian, China), cefoperazone-sulbactam (60 mg/kg q12h, Pfizer, Dalian China) and piperacillin-tazobactam (40 mg/kg q8h, Zhejiang Medicine Co., China; piperacillin was unavailable), respectively. If the symptom did not improve after 7-day of macrolides for therapy (included ambulatory treatment followed by hospitalization treatment of the same antibiotic), it would be considered as poor efficacy or inefficacy, and cefoperazone-sulbactam or piperacillin-tazobactam would be prescribed instead.

The study was conducted following the guidelines proposed in the Declaration of Helsinki and national and institutional standards (19). It has been approved by the Local Research Ethics Committee of the Children’s Hospital, Zhejiang University School of Medicine (2017-IRB-014). Written informed consent was obtained from the patients (> 8 years old) and their participants’ legal guardians for the study.

### Culture and identification for *Bordetella pertussis*

The collection of the nasopharyngeal swab, inoculation, and bacteria culture was performed as described previously (20). Briefly, clinical specimens were plated onto charcoal agar plates with cephalexin. Nasopharyngeal swabs made of calcium alginate (Copan, Italy) were used in the study, and charcoal agar (CM0119, Oxoid, United Kingdom) medium plates were prepared according to protocol.

### Minimum inhibitory concentrations determination of antimicrobial agents against *Bordetella pertussis*

Minimum inhibitory concentrations (MICs) of the following antibiotics were tested with *E*-test methods according to the protocol in previous study (20). The *E*-test strips of erythromycin (0.016–256 mg/L), azithromycin (0.016–256 mg/L), clindamycin (0.016–256 mg/L), sulfamethoxazole-trimethoprim (0.002–32 mg/L), ampicillin (0.016–256 mg/L), piperacillin (0.016–56 mg/L), ceftriaxone (0.002–32 mg/L), ceftazidime (0.016–256 mg/L), and meropenem (0.016–256 mg/L), were produced by bioMériex Ltd. (France). The quality control strains used were *Escherichia coli* ATCC 25922 and *Staphylococcus aureus* ATCC 29213.

### Statistical analysis

Statistical analysis was performed using SPSS 24.0 (Chicago, United States). Normal distribution data were shown as mean ± standard deviation (M ± *SD*). The group differences of normal distribution data were compared by *t*-test. Abnormal distribution data were presented as medians with inter quartile ranges (IQR). The group differences of abnormally distributed data were compared by the Mann-Whitney *U*-test. Enumeration data were described as numbers and percentages (*n*, %). The group differences in enumeration data were compared by the χ*^2^* test or Fisher’s exact test. We assessed the associated factors of positive pertussis using the univariate analyses and binary logistic analyses. A *p*-value < 0.05 was considered to be statistically significant.

## Results

### Characteristics of families included in the study

**FIGURE 1:**
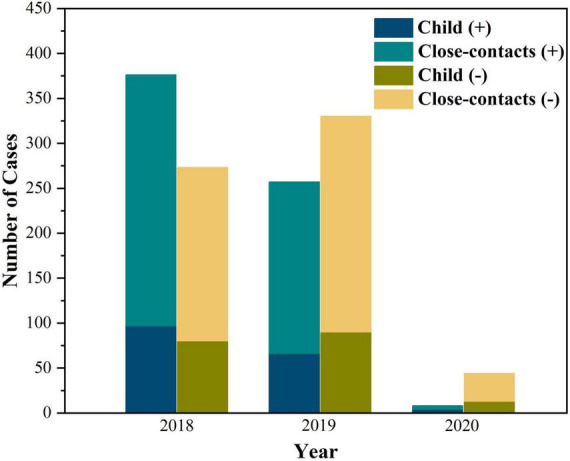
The yearly distribution of children and close contacts between 2018 and 2020.

Table 1 presents the results of the nasopharyngeal swabs culture test for *B. pertussis* in children with pertussis and their close contacts in households. A total of 350 index cases and 946 close contacts in households from 350 families were enrolled from January 1, 2018, to December 31, 2020 (Figure 1). The household close contacts included 350 mothers, 350 fathers, 115 grandmothers 64 grandfathers, 59 siblings, and 8 babysitters. Nasopharyngeal swabs were positive for *B. pertussis* based on culture in 130 (37.1%) index cases and 116 (12.3%) household close contacts. Among the 116 close contacts with positive culture results, 67 (57.8%) were mothers, 17 (14.7%) were fathers, 18 (15.5%) were grandmothers, four (3.4%) were grandfathers, eight (6.9%) were siblings and two (1.7%) were babysitters. Here, 103 (88.8%) were asymptomatic and 13 (11.2%, including 8 mothers, 3 fathers, 1 grandmother, and 1 7-year-old sister) had a persistent cough at inclusion. 167 out of 350 (47.7%) families had one or more members with positive cultural results for *B. pertussis*. To be noted, there was a family with four out of five members who tested positive. Namely, a total of 167 cases were diagnosed with pertussis, including 130 (37.1%, 130/350) index cases with positive culture results and 37 (10.7%, 37/350) from families having household close contacts with positive culture results. And the cases of all family members with negative culture were considered pertussis-like cases. Among all household close contacts, the positive rate of pertussis was higher in adult female members than that in adult male members (16.3% *vs.* 5.1%, χ*^2^* = 28.2, *P* < 0.01). And mothers (7.1%, 67/946) were found to be the most member of positive household close contacts.

**TABLE 1:** Distribution of nasopharyngeal swabs culture results for *B. pertussis* in 350 index cases and their household close contacts.

Household close contacts structure	Household close contacts *n* = 946	Index cases *n* = 350	
		
		*B. pertussis* positive *n* = 130	*B. pertussis* negative *n* = 220
Positive close-contacts	116	73	43
Mother	67	43	24
Father	17	11	6
Grandfather	4	2	2
Grandmother	18	13	5
Sibling	8	4	4
Baby-sitter	2	0	2
Negative close-contacts	830	302	528
Mother	283	87	196
Father	333	119	214
Grandfather	60	16	44
Grandmother	97	47	50
Sibling	51	27	24
Baby-sitter	6	0	6

There were 58 multi-child families and 292 one-child families. The positive rate for *B. pertussis* of index cases in the multi-child family (51.7%, 30/58) was significantly higher than that in one-child families (37.7%, 110/292, χ*^2^* = 3.982, *P* = 0.046); More positive families (had one or more than one member with positive culture result) were found in the multi-child family (60.3%, 35/58) when compare with one-child family (45.2%, 132/292, χ*^2^* = 4.446, *P* = 0.035).

### Demographic information and clinical features

**FIGURE 2:**
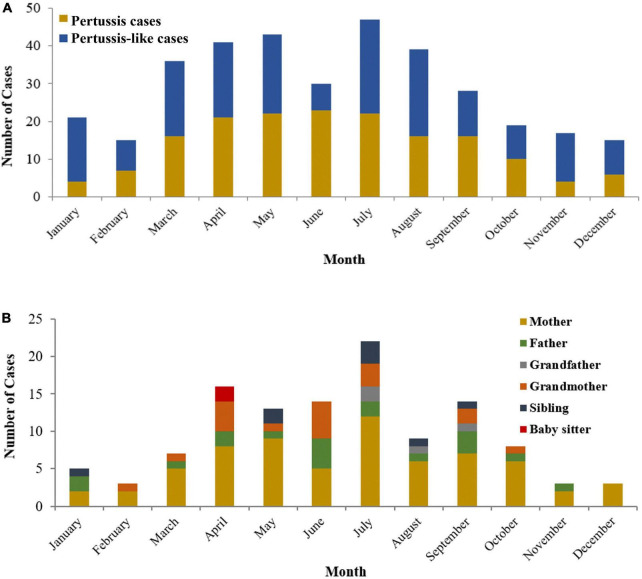
The distribution of pertussis cases **(A)** and positive household close-contacts **(B)** between 2018 and 2020.

The distribution of pertussis cases and their household close contacts between 2018 and 2020 is shown in Figure 2. The age of pertussis cases ranged from 21 days to 8.3 years old; 53.3% were under 6 months of age, and they were older than those in pertussis-like cases (5 months and 8 days vs. 4 months and 9 days, *Z* = -2.998, *P* = 0.003). No significant differences in sex(χ*^2^* = 0.064, *P* = 0.800) and season (χ*^2^* = 5.433, *P* = 0.143) were found between the two groups. The course of *B. pertussis* was isolated in 130 index cases from day 10 to day 71 after initial onset, with a median of 21 (IQR: 17–24) days. The incubation time for *B. pertussis* strains was 6 (IQR: 5–7) days. Of all pertussis cases, 48 (28.7%) were unvaccinated, and 76 (45.5%) were vaccinated with three or more doses of diphtheria-pertussis-tetanus, 38 (22.8%) had been vaccinated with one or two doses. Details on vaccination status and pre-antibiotics for therapy in pertussis cases and pertussis-like cases are shown in Table 2.

**TABLE 2:** Vaccination and antibiotic therapy status and clinical outcome of pertussis cases and pertussis-like cases.

		Pertussis cases *N* = 167	Pertussis-like cases *N* = 183	χ ^2^/Z	*P*
***Vaccination*** **status < 6 months children**					
Unvaccinated	*n* (%)	45 (26.9)	77 (42.1)	8.803	0.003
Vaccinated1-2doses	*n* (%)	30 (18.0)	40 (21.9)	0.827	0.363
Vaccinated ≥ 3 doses	*n* (%)	11 (6.6)	9 (4.9)	0.451	0.502
Unknown^a^	*n* (%)	3 (1.8)	2 (1.1)	0.673	0.457
Delayed vaccination	*n* (%)	32 (19.2)	44 (24.0)	0.998	0.318
***Antibiotic therapy before hospitalization***					
Yes	*n* (%)	158 (94.5)	173 (94.5)	0.001	0.975
Macrolides					
Cases	*n* (%)	69 (41.3)	70 (38.3)	0.343	0.558
Course (days)	Median (IQR)	4 (2.5,7)	4 (2,6)	–0.202	0.894
*Beta-lactams*					
Cases	*n* (%)	10 (6.0)	9 (4.2)	0.195	0.659
Course (days)	Median (IQR)	3 (1,5.25)	4 (2,6)	–0.788	0.447
Macrolides and β-lactams					
Cases	*n* (%)	66 (39.5)	70 (38.3)	0.059	0.808
Course (days)	Median (IQR)	8 (5,12)	12 (7,15)	–2.837	0.04
Others^b^	*n* (%)	13 (7.8)	24 (13.1)	2.624	0.105
***Outcome in*** **< *6 months children*** ^c^				–	–
Hospitalization	*n* (%)	89 (53.3)	125 (66.8)	8.283	0.004
Length of stay	Median (IQR)	14 (13,17)	9 (7,13)	–6.949	0.00

^a^Unknown refer to data loss due to the unavailability of information.

^b^Others refer to antibiotics other than macrolides and β-lactams.

^c^Among 167 pertussis cases, 89 were younger than 6 months old and all were inpatients. Of the 183 pertussis-like cases, 179 were hospitalized, and 125 were hospitalized in patients younger than 6 months of age.

Table 3 shows the differences in age, gender and family-related factors across subgroups of 350 index patients. Statistically significant differences were found in all factors except for gender (*P* < 0.05). Percentages of having other female caregivers (except for mothers) were higher in the households with at least one positive case (child and/or close contacts) than those with negative cases (child and close contacts) (*P* < 0.001). The similar pattern was also observed in terms of the household with four or more family members (*P* < 0.001).

**TABLE 3:** Differences in age, gender and family-related factors across subgroups (*n* = 350).

Characters		Child (–)/close contacts (–)	Child (–)/close contacts (+)	Child (+)/close contacts (–)	Child (+)/close contacts (+)	*P*-value
Age						0.011
	0–3 months	57 (31.1%)	7 (18.9%)	13 (19.4%)	17 (27.0%)	
	3–6 months	71 (38.8%)	8 (21.6%)	21 (31.3%)	24 (38.1%)	
	> 6 months	55 (30.1%)	22 (59.5%)	33 (49.3%)	22 (34.9%)	
Gender						0.777
	Male	100 (54.6%)	17 (45.9%)	37 (55.2%)	35 (55.6%)	
	Female	83 (45.4%)	20 (54.1%)	30 (44.8%)	28 (44.4%)	
Multiple children						0.078
	No	160 (87.4%)	32 (86.5%)	53 (79.1%)	47 (74.6%)	
	Yes	23 (12.6%)	5 (13.5%)	14 (20.9%)	16 (25.4%)	
Other female caregivers in the household except for mother						<0.001
	No	143 (78.1%)	21 (56.8%)	41 (61.2%)	30 (47.6%)	
	Yes	40 (21.9%)	16 (43.2%)	26 (38.8%)	33 (52.4%)	
Number of family members in the household						<0.001
	Three	122 (66.7%)	19 (51.4%)	30 (44.8%)	22 (34.9%)	
	Four or more	61 (33.3%)	18 (48.6%)	37 (55.2%)	41 (65.1%)	

Of all 167 pertussis cases, the cough duration at the time of enrollment ranged from 3 to 65 days with 64.1% reporting more than 14 days of cough; 160 (95.8%) had pertussis likes paroxysmal cough symptoms, 144 (86.2%) had a nocturnal cough, 101 (60.5%) had whooping, 54 (32.3%) had post-tussive vomiting, 48 (28.7%) had cyanosis, and 46 (27.5%) had wheezes. The moist rales were heard in 50 (29.9%) cases. Signs of condensation were seen on chest radiographs in 63 (40.8%) patients, including three with pleura reactions, one with pleural effusion, and two with localized emphysema. The top three co-colonizing bacteria were *Haemophilus influenza* (in 14 patients), *Streptococcus pneumonia* (in 8 patients), and *Glucococcus aureus* (in 5 patients). *Parainfluenza virus type 3* and *respiratory syncytial virus* were identified in 12 and seven patients, respectively. 58 (34.7%) had leukocytosis (> 20.0 × 10^9^/L) and 103 (61.7%) had lymphocytosis (> 10.0 × 10^9^/L). Before enrollment in the study, 158 out of 167 cases with pertussis (94.6%) had received antibiotic therapy, while 173 out of 183 cases with pertussis-like (94.5%) had received antibiotic therapy. The rate of antibiotic therapy was similar in both groups (*p* = 0.975). Macrolide treatment was used in 69 children (41.3%), among whom 60 children with a positive test result. Ten children (5.9%) received β-lactams treatment and 66 (39.5%) children received both β-lactams and macrolide treatment.

### Minimum inhibitory concentrations in antibiotics susceptibility test

Table 4 shows the results of MICs of the 9 antimicrobial agents in the 130 *B. pertussis* isolates. 98 out of 130 (75.4%) isolates had MICs of erythromycin, azithromycin, and clindamycin higher than 256 mg/L. MICs of piperacillin was < 0.016 mg/L in 127 out of 130 (97.7%) isolates. Macrolide-resistant *B. pertussis* isolates were identified in five patients who have not been prescribed any previous antibiotics, and fifteen patients who have not been prescribed any macrolide antibiotics during the whole treatment course of the disease.

**TABLE 4:** MICs of 9 antimicrobial agents against 130 *B. pertussis* strains isolated from index cases.

Antibiotics agents	MIC range (mg/L)	MIC_50_^a^ (mg/L)	MIC_90_^b^ (mg/L)
Erythromycin	0.047– > 256	>256	>256
Azithromycin	0.016– > 256	>256	>256
Clindamycin	0.023– > 256	>256	>256
SXT	0.016–1.000	0.190	0.50
Ampicillin	0.032–0.500	0.125	0.25
Piperacillin	<0.016–0.094	<0.016	<0.016
Ceftriaxone	0.008–0.125	0.064	0.094
Ceftazidime	0.023–1.000	0.940	0.19
Meropenem	0.006–0.125	0.047	0.125

MIC, minimum inhibitory concentration; SXT, Trimethoprim-sulfamethoxazole.

^a^MIC50, MIC at which 50% of the isolates tested are inhibited.

^b^MIC90, MIC at which 90% of the isolates tested are inhibited.

### Treatments and prognosis

All 167 children with pertussis were hospitalized and received anti-infective treatment with a median treatment duration of 14 days (IQR: 11–16 days). 117 cases (70.1%) were treated with a macrolide for a maximum duration of 14 days, of which 58.7% were switched to cefoperazone-sulbactam or piperacillin-tazobactam after 7-day of macrolides with poor efficacy. A total of 147 (88%) children were discharged from the hospital with significant improvement in symptoms such as paroxysmal cough, cyanosis, and post tussive vomiting after treatment. Thirty-four cases (20.4%) had aggravated coughing symptoms after 2 weeks of discharge. Table 5 presents the clearance rate of medications used to treat pertussis among 130 children. The clearance rate of *B. pertussis* in nasopharynx treated by cefoperazone-sulbactam or piperacillin-tazobactam after 1 and 2 weeks were 65% (13/20) and 90.0% (18/20), respectively, while the clearance rate of macrolides after 1 and 2 weeks were 41.7%(5/12) and 75.0% (9/12), respectively. There was no significant difference in the clearance rate between the two medications (1 week: χ*^2^* = 1.659, *P* = 0.198; 2 week: χ*^2^* = 1.280, *P* = 0.258).

**TABLE 5:** The clearance rate of *B. pertussis* in treatment with different antibiotics.

Antibiotics^a^	Cases (*n*)	Clearance rate in nasopharynx% (*n*)	
		
		1 week into treatment	2 weeks into treatment
Macrolides^b^	12	41.7 (5/12)	75.0 (9/12)
Cefoperazone-sulbactam	18	66.7 (12/18)	94.4 (17/18)
Piperacillin-tazobactam	2	50.0 (1/2)	50.0 (1/2)
Cefoperazone-sulbactam instead of macrolides with poor efficacy^c^	64	59.4 (38/64)	92.2 (59/64)
Piperacillin-tazobactam instead of macrolides with poor efficacy^c^	14	71.4 (10/14)	92.9 (13/14)
Others	20	25.0 (5/20)	80.0 (16/20)
Total	130	54.6 (71/130)	88.5 (115/130)

^a^Besides antibiotics in hospitalization, ambulatory treatment was included when the same antibiotic was continued after admission.

^b^Seven with erythromycin, five with azithromycin.

^c^If the symptom did not improve after 7-day of macrolides for therapy (included ambulatory treatment followed by hospitalization treatment of the same antibiotic), it would be considered as poor efficacy or inefficacy, and cefoperazone-sulbactam or piperacillin-tazobactam would be prescribed instead.

A total of 130 patients were followed up for 4 weeks and found that *B. pertussis* strains isolated from index cases turned negative with a median time of 7 days (IQR:7–14 days). To be noted, in three cases, nasopharyngeal swab culture was negative for 2–3 weeks and then re-cultured to *B.* pertussis.

## Discussion

Infection of *B. pertussis* occurs more frequently in children, and the clinical manifestations of pertussis in children are more severe than in adults. Pertussis is characterized by cluster infection and is commonly found gathering in families and schools. In our study, the median age of pertussis patients was 5 months and 8 days, and around 60% of pertussis cases had at least one household close contact with positive culture results. Given the children’s simple life path and minimal contact with the external environment, the infected household’s close contacts could be considered the main source of pertussis infection (21). Moreover, the *B. pertussis* culture-positive rate in adult female family members was higher than that in adult male family members. Since the child is mainly taken care of by their mothers or grandmothers given the caring pattern in Chinese families, the child usually has closer contacts with the female caregivers. However, due to the mild or asymptom of most adults infected with pertussis, it is difficult to trace the source and to distinguish the specific transmission pathway whether adults infect children or children who were infected in the nursery or school then transmitted to other family members.

In the present study, the pertussis cases with culture-negative for *B. pertussis* in children but positive in their close contacts in the household accounted for 10.6%. The potential explanation may be the use of antimicrobial that can significantly affect the positive rate of pertussis culture. We suggested to test *B. pertussis* culture for both children and close contacts in the household which can identify all the positive pertussis cases in one household, so the physicians can make a reasonable treatment plan for the households at the same time.

In this study, the children frequently had severe symptoms while the adult household close contacts usually had mild or asymptom, which was consistent with the previous studies in Canada (22). The *B. pertussis* isolated in one family should be considered as the same clone and homogeneity, and the reason for the difference in clinical symptoms between children and adults may be that adults usually have partial immunity due to previously mild or asymptomatic infection (23, 24).

As a seasonal infectious disease, the pertussis cases recruited herein mainly occurred from April to September in Zhejiang Province, China, which was consistent with our previous research results (20). And we found an obvious reduction in pertussis patients during COVID-19 in 2020, which was consistent with the previous study also in Zhejiang Province (25). It might be ascribed to the long-term suppression effect from the promotion of masks and school closures stage.

Our data showed that the infants were the predominant patients and were all hospitalized, which was related to the severe clinical symptoms and high attendance rate of infants. According to the Chinese immunization program, infants should receive diphtheria, tetanus and acellular pertussis combined vaccine (DTap) at 3, 4, and 5 months or adsorbed diphtheria- tetanus-acellular pertussis-inactivated poliovirus-Haemophilus influenza type b combination vaccine (DTaP-IPV/Hib) vaccine at 2, 3, 4 months, and received a booster vaccine at 18–24 months. Among the pertussis cases in this study, most of the children were less than 6 months old and had not completed the basic pertussis vaccine immunization, but 6.6% (11/167) of the pertussis cases aged < 6 months had been vaccinated with three doses of the DTap vaccine. Since 2007, co-purified acellular pertussis vaccines (APVs) have been included in the Chinese national EPI. Whole-cell pertussis vaccines (WPVs) was replaced by APVs completely in 2009 in Hangzhou and in 2013 throughout the country (26). In our study, all patients were born after 2010, so most of them received APVs except a few from other cities. Coincidentally, a dramatic increase of pertussis cases was found between 2013 and 2018 after WPVs vaccine being switched to APVs in China (27). Thus, we think that there might be connection between the re-emergence of pertussis and the switch of vaccine. Although *B. pertussis* is a pathogen of the upper respiratory tract, we found quite a lot of patients occurred pneumonia in our study, which was consistent with some previous studies (20). It may be related to the children prone to co-infection with *Haemophilus influenza* and *Parainfluenza virus type 3* and other pathogens (28, 29).

The first recommended antibiotic for pertussis treatment remains macrolides, such as erythromycin and azithromycin. However, MRBP has increased in recent years in some countries, especially in some regions of China (16). The macrolides-resistant rate in the mainland China has been over 50%, which is rare in European countries and the US. In particularly, despite the use of macrolides for pertussis for 60 years in the US, only < 0.5% of the total *B. pertussis* examined have been detected as MRBP from 1994 to 2020 (30). Our results showed that 75.4% of the isolates had MICs of erythromycin, azithromycin, and clindamycin greater than 256 mg/L, which was consistent with previous findings in southern China (57.4–75.4%) (20, 28). Meanwhile, a higher prevalence of erythromycin-resistant *B. pertussis* was also found in western and northern China (79.3–91.9%) (27). The high drug resistance should be the main cause of the failure of macrolides treatment. With the low MICs and the strong ability to clear *B. pertussis* strains that existed in the nasopharynx, cefoperazone-sulbactam and piperacillin have been the alternative antibiotics to treat macrolide-resistant pertussis (20), their anti-infection course is advised as 2 weeks as that of macrolides. However, in this study, there was no significant difference in the clearance rate for *B. pertussis* by cefoperazone-sulbactam or piperacillin-tazobactam for 1 and 2 weeks compared with macrolides. It may be related to the insufficient number of specimens. Given the low MICs of SMZ, it is suitable for anti-infective and prophylactic treatment in elderly children and adults. All carriers with *B. Pertussis* in nasopharynx are sources of contagiosity. There were no Chinese recommendations for treatment of pertussis-contacts. In this study, all of the positive contacts were advised to be treated with antibiotics: Oral SMZ 0.96 g, twice a day for 7–14 days (for those without severe symptoms); or intravenous cefoperazone-sulbactam for 7 days followed by oral SMZ (for those without severe symptoms). Unfortunately, many positive adult-contacts were not prescribed with advised antibiotics when they visit a doctor in adult hospital. In terms of antibiotic prophylaxis, close contact in the household, exposure to high-risk diseases, or close contact with high-risk groups should be presented within 21 days of contact (31). The protection provided by the pertussis-specific IgG antibodies from a mother who has been vaccinated during pregnancy is considered the primary strategy. If this strategy does not work, a “cocoon strategy” is recommended, in which close contacts of infants and young children under 6 months of age are vaccinated to form a protective circle around them (32).

The differences in drug resistance patterns between China and other countries may be related to the historical exposure to macrolides. Meanwhile, macrolide resistance may also be underestimated as PCR is routinely performed in some countries instead of bacterial culture and antimicrobial susceptibility. Bacterial culture is conducive to investigating the drug resistance patterns and remains the “gold standard” for pertussis diagnosis. As is known, the positive rate of *B. pertussis* culture is higher in the early stage of pertussis infection, especially for the patients without antibiotic treatment (33). However, the children in this study generally had a long course of the disease. In one case, *B. pertussis* even could be isolated from the patient up to more than 70 days after the initial onset of cough. Therefore, a bacterial culture is recommended for the children suspected of whooping cough when conditions are available, regardless of the duration of the disease. The bacterial culture process is safe in the context of the proper personal protection and specimen handling of specimens. *B. pertussis* culture has been carried out in our hospital for 6 years without any laboratory infection. As the pertussis DNA testing kit has not been approved in China until 2021, it cannot be carried out with DNA testing at the same time.

The main limitations of this study were that the diagnosis of pertussis was only based on the bacterial culture without PCR test. False-negative cases may also be present in individuals who have been treated with antibiotics. We consider that some of the patients with negative results might have pertussis. Without bacteriological confirmation in absence of PCR, they were diagnosed as pertussis-like syndrome. In addition, *B. pertussis* antibody detection was not performed for children during the recovery period to evaluate the immune protective effect after infection. Another limitation is that this study lacks the historical context necessary to trace the arc of pertussis in family clusters. We recommended to carry out pertussis culture, PCR, and ELISA simultaneously to dynamically monitor the infection rate of pertussis among children and their household close contacts.

## Conclusion

In conclusion, pertussis is a common infectious disease mainly occurred in children, especially in infants under 6 months of age. Close contacts in households are prone to cluster infection. In particular, the household with multiple children, having other female caregivers except for mothers and four or more family members at home could increase the risk of pertussis. Culture for *B. pertussis* both in children and their close contacts can improve the diagnosis rate of pertussis in children. Isolates of *B. pertussis* in China are highly resistant to macrolides.

## Data availability statement

The original contributions presented in the study are included in the article/Supplementary material, further inquiries can be directed to the corresponding author/s.

## Ethics statement

The studies involving human participants were reviewed and approved by the Ethics Committee and the Institutional Board of Privacy and Security at the hospital (2017-IRB-014). Written informed consent to participate in this study was provided by the participants’ legal guardian/next of kin.

## Author contributions

C-ZH and L-NL: conceptualization. C-ZH, L-NL, and J-SZ: methodology. C-ZH, L-NL and G-NB: formal analysis, data curation, visualization, writing—original draft preparation, writing, review, and editing. L-NL and J-SZ: investigation. C-ZH, Y-MM and M-MZ: resources. C-ZH and G-NB: supervision. C-ZH: project administration and funding acquisition. All authors contributed to the article and approved the submitted version.
