# Supplementary material for: Epidemiological and clinical characteristics of pertussis in children and their close contacts in households: A cross-sectional survey in Zhejiang Province, China
Source: Front Pediatr. 2022 Aug 18;10:976796. doi: 10.3389/fped.2022.976796 (PMC9434343; doi:10.3389/fped.2022.976796)
Supplement: Supplementary file 1 [file Data_Sheet_1.PDF]

Supplementary Material

Epidemiological and clinical characteristics of pertussis in children and their close contacts in households: a cross-sectional survey in Zhejiang Province, China

Luo-Na Lin[1+], Jin-Si Zhou[1+], Chun-Zhen Hua[1*], Guan-Nan Bai[1], Yu-Mei Mi[1], Ming-Ming Zhou[2]

[1] Division of Infectious Disease, Children's Hospital, Zhejiang University School of Medicine, National Center for Clinical Medicine Research in Children's Health and Disease, Hangzhou 310003, China

[2] Department of Clinical Laboratory Center, Children's Hospital, Zhejiang University School of Medicine, Hangzhou 310003, China

[*]**Correspondence:**

Chun-Zhen Hua

huachunzhen@zju.edu.cn

[+]These authors have contributed equally to this work

Table S1. Overview of studies of pertussis in adolescents and adults in China

Citation	Period	City	Age (year)	N	Serological cut-off value (PT IgG)	PT-IgG positivity rate (%)	Estimated pertussis infection rates
[1]	2019	Ningbo	1-91	1206	≥40 IU/ml	Peaks: ≥ 5 years 7.6%<br>≥ 50years7.3%	9302/100,000<br>8967/100,000
[2]	2018-2019	Chongqi	0-76	666	≥40 IU/ml	Age years (n):<br>6–18 (87): 4.6%<br>18-40 (74):5.4%<br>40-50 (62): 3.2%<br>50-60 (61):6.6%<br>≥60 (79):7.6%	5644/100,000<br>6626/100,000<br>3926/100,000<br>8098/100,000<br>9325/100,000
[3]	Apr.-Jun. 2017	Beijing	0-76	2144	≥40 IU/ml	Peaks: 15 years 8.3%<br>45 years 8.6%	10,184/100,000<br>10,552/100,000

Table S1 continued

Citation	Period	City	Age (year)	N	Serological cut-off value (PT IgG)	PT-IgG positivity rate (%)	Estimated pertussis infection rates
[4]	2010-2016	Beijing	2-69	3058	≥40 IU/ml	Age years (n):<br>10–19 (26): 5.8%<br>20-29 (25): 5.5%<br>30-39 (21): 4.9%<br>40-49 (28): 6.1%<br>50-59 (14): 3.4%<br>60-69 (27): 6.6%	7117/100,000<br>6749/100,000<br>6012/100,000<br>7485/100,000<br>4172/100,000<br>8098/100,000
[5]	Oct.-Nov. 2015	Chongqi	1-59	1080	≥100 IU/ml	Peaks: 7-14years: 1.6%<br>≥20 years: 2.2%	9971/100,000<br>13,898/100,000

PT-IgG: IgG antibody against pertussis

Table S2. The comparison of macrolide resistant rate in the present study and in different Chinese studies

Region	Period	Macrolide resistant rate	Reference
**Northern**			
Beijing,Hebei, Shangdong,Shanxi,Tianjing et al.	2013-2014	91.9% (91/99)	[6]
Beijing,Hebei, Shangdong,Shanxi,Tianjing et al.	2014-2016	91.1% (194/213)	[7]
**Western**			
Xi’an	2012-2013	87.5% (14/16)	[8]
Xi’an	2018-2020	79.3% (46/58)	[9]
**Southern**			
Guangdong	2014-2016	64.3% (36/56)	[7]
Shenzhen	2015-2017	48.6% (51/105)	[10]
Hunan	2017-2018	49.1% (27/55)	[11]
**Eastern**			
Shanghai	2016-2017	57.4% (81/141)	[12]
Zhejiang	2016	75.4% (95/126)	[13]
Zhejiang	2016-2018	62.4% (78/125)	[14]
Zhejiang	2018-2020	75.4% (98/130)	This work

References

[1] Zhao XF, Ye S, Ma R, Dong HJ, Fang T, Xu GZ. Seroepidemiology of pertussis in healthy population in Ningbo, 2019. Zhonghua Liu Xing Bing Xue Za Zhi. (2021) 42(4):638-642. doi: 10.3760/cma.j.cn112338-20200629-00894

[2] Liu D, Cheng X, Wei S, Yuan L, Chen C, Yao K. Decline of serologic immunity to diphtheria, tetanus and pertussis with age suggested a full life

vaccination in mainland China. Hum Vaccin Immunother. (2021)17(6):1757-1762. doi:10.1080/21645515.2020.1840253
[3] Zhang Z, Pan J, Chen M, Zhang T, Li J, Lu L. Seroepidemiology of pertussis in China: A population-based, cross-sectional study. Vaccine. (2021)39(12):1687-1692. doi: 10.1016/j.vaccine.2021.02.032.
[4] Zhang Y, Chen Z, Zhao J, Zhang N, Chen N, Zhang J, et al. Increased susceptibility to pertussis in adults at childbearing age as determined by comparative seroprevalence study, China 2010-2016. J Infect. (2019)79(1):1-6. doi: 10.1016/j.jinf.2019.04.011
[5] Yao N, Zeng Q, Wang Q. Seroepidemiology of diphtheria and pertussis in Chongqing, China: serology-based evidence of Bordetella pertussis infection. Public Health. (2018) 156:60-66. doi: 10.1016/j.puhe.2017.12.009.
[6] Yang Y, Yao K, Ma X, Shi W, Yuan L, Yang Y. Variation in Bordetella pertussis susceptibility to erythromycin and virulence-related genotype changes in China (1970-2014). PLoS One. (2015) 10(9): e0138941. doi: 10.1371/journal.pone.0138941
[7] Li LJ, Deng JK, Ma X, Zhou K, Meng QH, Yuan L, et al. High prevalence of macrolide-resistant Bordetella pertussis and ptxP1 genotype, mainland China, 2014-2016. Emerg Infect Dis. (2019) 25(12): 2205-2214. doi:10.3201/eid2512.181836
[8] Wang Z, Cui Z, Li Y, Hou T, Liu X, Xi Y, et al. High prevalence of erythromycin-resistant Bordetella pertussis in Xi'an, China. Clin Microbiol Infec. (2014) 20(11): 0825-0830. doi: 10.1111/1469-0691.12671
[9] Zhang J, Zhang D, Wang X, Wei X, Li H. Macrolide susceptibility and molecular characteristics of Bordetella pertussis. J Int Med Res. (2022)50(2): 3000605221078782. doi: 10.1177/03000605221078782.
[10] Zhang JS, Wang HM, Yao KH, Liu Y, Lei YL, Deng JK, et al. Clinical characteristics, molecular epidemiology and antimicrobial susceptibility of

pertussis among children in southern China. World J Pediatr. (2020)16(2): 185-192.doi: 10.1007/s12519-019-00308-5

[11] Lin XJ, Zou J, Yao KH, Li LJ, Zhong LL. Analysis of antibiotic sensitivity and resistance genes of Bordetella pertussis in Chinese children. Medicine. (2021)100(2): e24090. doi: 10.1097/MD.0000000000024090

[12] Fu P, Wang C, Tian H, Kang Z, Zeng M. Bordetella pertussis infection in infants and young children in Shanghai, China, 2016-2017: clinical features, genotype variations of antigenic genes and macrolides resistance. Pediatr Infect Dis J. (2019) 38(4): 370-376. doi:10.1097/INF.0000000000002160

[13] Hua CZ, Wang HJ, Zhang Z, Tao XF, Li, JP , Mi, YM, et al. In vitro activity and clinical efficacy of macrolides, cefoperazone-sulbactam and piperacillin/piperacillin-tazobactam against Bordetella pertussis and the clinical manifestations in pertussis patients due to these isolates: a single-centre study in Zhejiang Province, China. J Glob Antimicrob Resist. (2019) 18:47-51. doi: 10.1016/j.jgar.2019.01.029

[14] Mi YM, Hua CZ, Fang C, Lu JJ, Xie YP, Lin LN, et al, Effect of macrolides and β-lactams on clearance of Bordetella pertussis in the nasopharynx in children with whooping cough. Pediatr Infect Dis J. (2021) (40)2: 87-90. doi: 10.1097/INF.0000000000002911.
